# High-resolution silicon photonic sensor based on a narrowband microwave photonic filter

**DOI:** 10.1007/s12200-023-00059-2

**Published:** 2023-03-27

**Authors:** Haiyan Luo, Lu Xu, Jie Yan, Qiansheng Wang, Wenwu Wang, Xi Xiao

**Affiliations:** 1 Institute of Microelectronics, Chinese Academy of Sciences, Beijing, 100029 China; 2 University of Chinese Academy of Sciences, Beijing, 100049 China; 3 National Information Optoelectronics Innovation Center, China Information and Communication Technologies Group Corporation, Wuhan, 430074 China

**Keywords:** Micro-ring resonator, Microwave photonic filter, Silicon photonics, Microwave photonic sensor

## Abstract

Microwave photonic sensors are promising for improving sensing resolution and speed of optical sensors. In this paper, a high-sensitivity, high-resolution temperature sensor based on microwave photonic filter (MPF) is proposed and demonstrated. A micro-ring resonator (MRR) based on silicon-on-insulator is used as the sensing probe to convert the wavelength shift caused by temperature change to microwave frequency variation via the MPF system. By analyzing the frequency shift with high-speed and high-resolution monitors, the temperature change can be detected. The MRR is designed with multi-mode ridge waveguides to reduce propagation loss and achieves an ultra-high Q factor of 1.01 × 10^6^. The proposed MPF has a single passband with a narrow bandwidth of 192 MHz. With clear peak-frequency shift, the sensitivity of the MPF-based temperature sensor is measured to be 10.22 GHz/°C. Due to higher sensitivity and ultra-narrow bandwidth of the MPF, the sensing resolution of the proposed temperature sensor is as high as 0.019 °C.

**Graphical Abstract:**

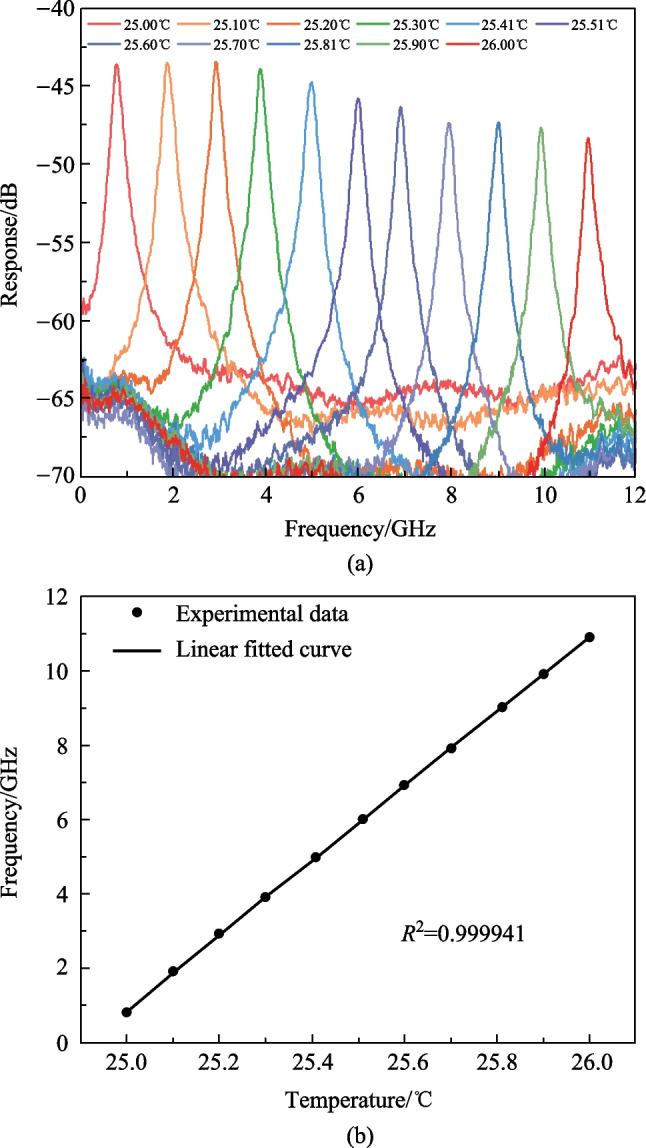

## Introduction

Over recent years, optical fiber sensors have been extensively investigated for their intrinsic advantages of low insertion loss, light weight, small size, high sensitivity, immunity to electromagnetic interference, and high tolerance to harsh environments [1]. Optical fiber sensors have been widely used in many applications such as temperature measurement, environmental monitoring, gas sensing, and biological sensing [2–6]. In conventional optical sensors, the sensing information is usually related to a wavelength shift, which is detected by an optical spectrum analyzer (OSA). The use of OSA with poor resolution and low interrogation speed limits the sensing resolution as well as the sensing speed of the optical fiber sensors.

Microwave photonics is a promising technology to solve these problems since it combines the advantages of optics with microwaves. Microwave photonic sensors (MPS) can convert the variation in the optical domain to a resulting change in the microwave domain, where the sensing information can be monitored with high speed and high resolution using electrical monitors and analyzers. Higher interrogation speed can also be achieved with the help of a digital signal processor (DSP). As a result, MPS has attracted increasing interest and a variety of high-performance MPS devices have been proposed [7–12]. The center frequency of microwave photonic filters (MPFs) can usually be widely tuned, which can be used to monitor the changes that the system senses. By directly mapping the optical wavelength shift to the microwave frequency variation, a minor change in the optical domain caused by the perturbation to be measured will be converted into a relatively large variation in the microwave domain, thus to improve the sensing speed and sensing resolution [13–16].

Integrated photonics based on silicon-on-insulator (SOI) has been rapidly developed and widely applied in implementing integrated MPFs, due to its high performance and compatibility with complementary metal oxide semiconductor (CMOS) processes. Compared with other materials such as SiO_2_ and Si_3_N_4_, which are also widely used for integrated photonics, silicon has a higher thermo-optic coefficient of 1.86 × 10^−4^/°C [17, 18]. Therefore, MPF-based temperature sensors implemented with high-Q factor micro-ring resonator (MRR) and microdisk resonator (MDR) on silicon platforms usually have higher sensitivity and higher resolution [19–22]. In 2018, Li et al. [19] realized a high-performance temperature-sensing probe based on a narrowband microwave photonic notch filter. The MPF was implemented with SOI-based reflective MRR and the sensitivity was 11.57 GHz/°C. In 2018, Liu et al. [20] proposed an on-chip optical sensor based on a dual-passband MPF incorporating a silicon photonic integrated MDR. Two whispering gallery modes of the MDR were employed to simultaneously measure the changes in temperature and refractive index. In the same year, Deng et al. [22] proposed an MPF-based temperature sensor with silicon photonic MDR. The high-Q factor MDR was used to sense the environmental change and converted it to microwave frequency shift by the incorporated MPF with a sensitivity of 9.6 GHz/°C.

In this paper, a high-resolution temperature sensor implemented with an ultra-high Q factor MRR is proposed and experimentally demonstrated. The MRR is designed with ultra-low loss waveguides and fabricated on the SOI platform, with the Q factor measured to be 1.01 × 10^6^. The proposed MPF is mainly composed of a laser source, a phase modulator (PM), an MRR, and a photodetector (PD). When sensing a temperature variation, the resonance of the MRR will have a wavelength shift, and the center frequency of the MPF will change accordingly. By detecting this center frequency change, a temperature sensor is implemented and the sensitivity is measured to be 10.22 GHz/°C. Due to the ultra-high Q factor of the MRR, the proposed single passband MPF has a narrow bandwidth of 192 MHz. Since the sensing resolution is associated with the bandwidth of the MPF and the sensitivity, the sensing resolution of the proposed temperature sensor is as high as 0.019 °C.

## Principle

### Principle of the MPF

**Fig. 1:**
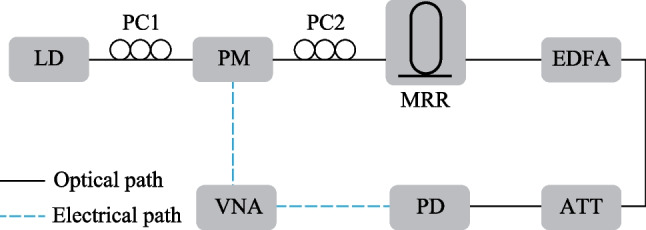
Schematic diagram of the proposed MPF-based sensor system. *LD* laser diode, *PC* polarization controller, *PM* phase modulator, *MRR* micro-ring resonator, *EDFA* erbium-doped fiber amplifier, *ATT* attenuator, *PD* photodetector, *VNA* vector network analyzer

The schematic diagram of the proposed MPF-based sensor system is shown in Fig. 1. The optical carrier is emitted from a laser diode. After polarization adjustment, the optical carrier is sent into a PM for modulation. The modulated signal is filtered by an MRR and then amplified by an erbium-doped fiber amplifier (EDFA). After adjusting the power to an appropriate level via an attenuator, the optical signal is received by a photodetector and the whole optoelectronic circuit is analyzed with a vector network analyzer (VNA).

When the optical carrier passes through the PM, the electrical field of the modulated signal can be expressed as

1 $$E_{{{\text{PM}}}} = E_{0} \cos \left[ {\omega_{0} t + {\uppi }\frac{{V_{{{\text{RF}}}} \cos (\omega_{{\text{m}}} t)}}{{V_{{\uppi }} }}} \right],$$

where *E*_0_ is the amplitude of the optical carrier, *V*_RF_ is the voltage of the modulation signal, *V*_π_ is the half-wave voltage of the modulator, *ω*_0_ and *ω*_m_ are the angular frequencies of the optical carrier and the modulation signal, respectively. Under small signal modulation, the amplitudes of the higher-order sidebands are usually much lower than those of the 1st-order sidebands and can be neglected. Equation (1) can be expanded with the Bessel function of the first kind as

2 $$E_{{{\text{PM}}}} { = }E_{0} \left\{ {J_{0} (m)\cos (\omega_{0} t) + J_{1} (m)\cos \left[ {(\omega_{0} + \omega_{{\text{m}}} )t + \frac{{\uppi }}{{2}}} \right] + J_{ - 1} (m)\cos \left[ {(\omega_{0} - \omega_{{\text{m}}} )t - \frac{{\uppi }}{{2}}} \right]} \right\},$$

where *m* = π*V*_RF_/*V*_π_ represents the modulation index, and *J*_*n*_ is the *n*th-order Bessel function of the first kind. With *J*_1_ =  − *J*_−1_, the current output by the PD can be expressed as

3 $$i_{{{\text{AC}}}} = \Re \left| {E_{0} } \right|^{2} \left[ {J_{0} (m)J_{1} (m)\cos \left( {\omega_{{\text{m}}} t + \frac{{\uppi }}{{2}}} \right) + J_{0} (m)J_{1} (m)\cos \left( {\omega_{{\text{m}}} t - \frac{{\uppi }}{{2}}} \right)} \right],$$

where ℜ is the responsivity of the PD. Equation (3) shows that the output consists of two beat signals. One is the beat signal between the + 1st-order sideband and the carrier, while the other is the beat signal between the − 1st-order sideband and the carrier. Without optical filtering, the two beat signals will be cancelled by each other and negligible output will be detected at the corresponding frequency. By introducing an optical filter to change the amplitude of one sideband at the angular frequency of *ω*_m_, the two beat signals will not be cancelled by each other and a single passband will be generated. In the spectrum of the microwave frequencies, a bandpass MPF with a center angular frequency of *ω*_m_ will be generated as a result.

### Design and fabrication of the ultra-high Q MRR

SOI-based MRRs are ideal optical filters for implementing MPFs since the advantages of MRR can improve the corresponding performance of the realized MPF. For instance, MRRs with a small footprint, high Q factor, and thermal sensitivity offer reduced size, precise frequency selectivity, and tunable center frequency for the realized MPFs [23, 24]. The common size of an SOI waveguide has a width of 500 nm and a height of 220 nm, which ensures single-mode transmission. The propagation loss of such a waveguide is about 2 dB/cm and the Q factor of the fabricated MRR is limited. The dominant loss of the ridge Si waveguide is typically the scattering loss at etched sidewalls [25]. As a result, by introducing multi-mode ridge waveguides, the fundamental TE mode can be confined in the central region of the waveguides and the scattering loss can be significantly reduced. As the propagation loss can be greatly decreased, an ultra-high Q MRR can be fabricated. To ensure single-mode transmission in the output port of the MRR, single-mode waveguides are used in the coupling and bending regions while linear adiabatic tapers are used for connecting the waveguides with different widths.

**Fig. 2:**
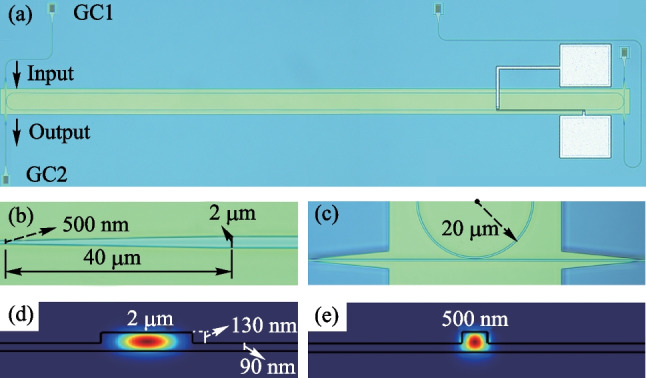
Micrographs of the fabricated MRR and simulated power distribution of the fundamental TE mode of MRR. **a** Global view of the MRR (GC: grating coupler). **b** Zoom-in view of the linear taper. **c** Zoom-in view of the coupling region. Simulated power distribution of the fundamental TE mode for **d** the 2 μm width ridge waveguide and **e** the 500 nm width ridge waveguide

The multi-mode and single-mode ridge waveguides are designed with widths of 2 μm and 500 nm, respectively. The ridge height is 130 nm while the slab height is 90 nm. The simulated results of the fundamental transverse electric (TE) mode of the multi-mode and single-mode ridge waveguides are shown in Fig. 2d, e, respectively. The taper with linearly changed width has a length of 40 μm while the radius of the bent waveguide is 20 μm. The designed MRR is fabricated with the standard CMOS-compatible SOI process at IME, Singapore. The micrographs of the fabricated MRR are shown in Fig. 2. Two grating couplers (GC) are connected to the input and through ports of the MRR to couple light into and out of the device.

### Simulation of the MPF-based temperature sensor

**Fig. 3:**
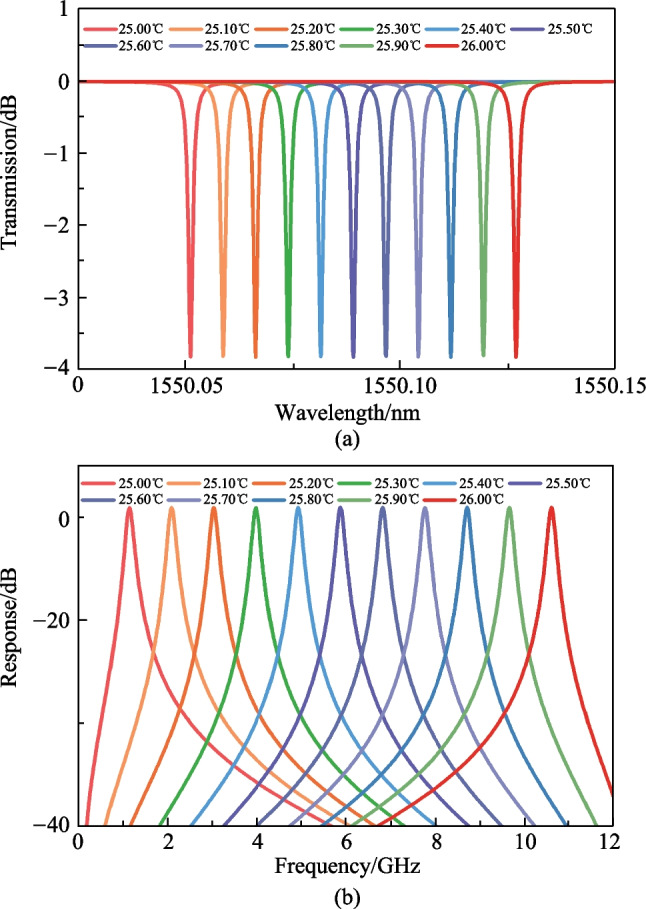
Simulated results of **a** the transmission spectra of the MRR and **b** the responses of the MPF with changed temperature

Since silicon has a relatively high thermo-optic coefficient of 1.86 × 10^−4^/°C [17], the SOI-based MRR will achieve high sensitivity as a temperature-sensing probe. The sensitivity of the MRR can be expressed as [26]

4 $$S = \frac{{{\text{d}}\lambda_{{{\text{res}}}} }}{{{\text{d}}T}} = \frac{{\lambda_{{{\text{res}}}} }}{{n_{{\text{g}}} }}\left( {\frac{{\partial n_{{{\text{eff}}}} }}{\partial T} + n_{{{\text{eff}}}} \alpha_{{{\text{Si}}}} } \right),$$

where *λ*_res_ is the resonant wavelength of the MRR, *n*_eff_ is the effective refractive index of the waveguide, *n*_g_ is the group refractive index of the waveguide and *n*_g_ = *n*_eff_ − *λ*· (*∂n*_eff_ /*∂λ*), *α*_Si_ is the thermal expansion coefficient of silicon. Here *α*_Si_ (2.5 × 10^−6^/°C [27]) can be ignored since it is two orders of magnitude smaller than the thermo-optic coefficient. With *n*_g_ ≈3.609 at wavelength of 1550 nm in the multi-mode ridge waveguide, the sensitivity of the MRR is roughly calculated as 76.27 pm/°C according to Eq. (4). By applying the corresponding refractive index at different temperatures, the transmission spectra of the MRR at different temperatures could be simulated and the results are shown in Fig. 3a. When the temperature changes from 25.00 °C to 26.00 °C in steps of 0.10 °C, the resonant wavelength of the MRR varies linearly from 1550.0512 to 1550.1268 nm with a total change of 0.0756 nm, indicating a corresponding sensitivity of 75.6 pm/°C. This agrees well with the calculated sensitivity. According to Eq. (3), the variation of resonant wavelength will also change the center frequency of the proposed MPF, and the simulated results are shown in Fig. 3b. With the same temperature change, the center frequency of the bandpass MPF varies from 1.149 to 10.590 GHz, indicating a sensitivity of 9.441 GHz/°C.

## Experimental results

To validate the proposed MPF-based temperature sensor, a proof-of-concept experiment is carried out. The experimental setup is illustrated as shown in Fig. 1. A continuous-wave (CW) light emitted from a laser diode (Santec TSL-570) is launched into the PM (iXblue MPZ-LN-40) after polarization controller 1 (PC1). The CW light is then modulated by the microwave signal generated from the VNA (Ceyear 3672E) via the PM. After polarization adjustment by PC2, the modulated signal is coupled into the input port of the MRR via GC1 (shown in Fig. 2) and then filtered by the MRR. The filtered signal at the through port of the MRR is coupled out from the chip via GC2 (shown in Fig. 2). After being amplified by an EDFA (Amonics AEDFA-33-R-FC), the output signal is power-adjusted by an optical attenuator and then detected by the PD (Finisar XPDV2320R). The whole chip is placed on a thermo-electric cooler (TEC) to monitor and control the temperature of the MRR. The output microwave signal is received by the VNA and the transmission of the proposed MPF is then analyzed.

**Fig. 4:**
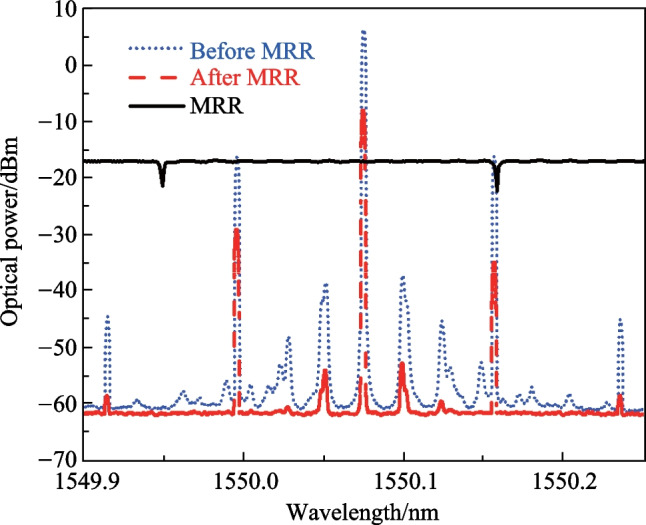
Measured optical spectra. The black solid curve shows the transmission of the MRR. The blue dotted curve and the red dashed curve show the phase modulated signals before and after the MRR, respectively

**Fig. 5:**
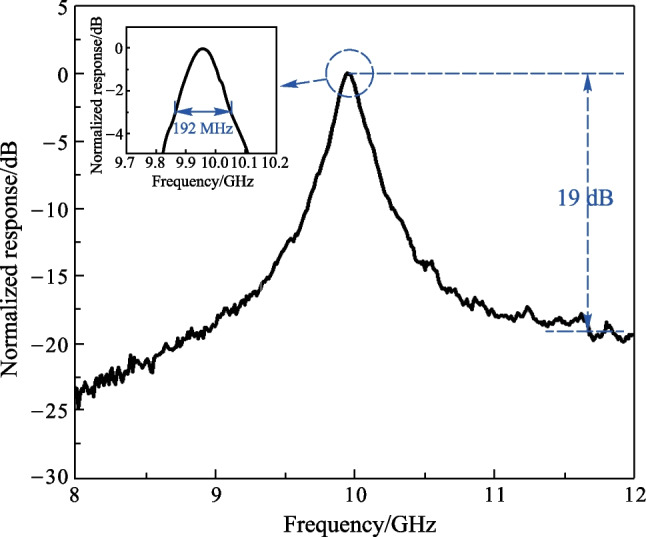
Normalized response of the proposed single passband MPF

The optical signal after phase modulation is measured by an OSA (APEX AP2087A) and the result is shown as a blue dotted curve in Fig. 4. The center wavelength is measured to be 1550.076 nm and the ± 1st-order sidebands are 0.08 nm from the carrier. The power of ± 2nd-order sidebands are 20 dB lower than that of ± 1st-order sidebands and can be neglected. The spectrum of the MRR is also measured and shown as a black solid curve in Fig. 4. The free spectral range (FSR) of the MRR is 0.209 nm. By controlling the temperature of the TEC, one of the resonant wavelengths of the MRR is adjusted to align with the − 1st-order sideband and the filtered optical signal is shown as a red dashed curve in Fig. 4. After filtering, the power of the − 1st-order sideband is lower than that of the + 1st-order sideband. According to Eq. (3), the beat signal between the − 1st-order sideband with the carrier and that between the + 1st-order sideband with the carrier will not be cancelled by each other. At other frequencies, the corresponding beat signals have the same power but opposite phase and are cancelled by each other as a result. A bandpass MPF will be generated and the result measured by the VNA is shown in Fig. 5. As seen in the inset of Fig. 5, the full width at half maximum (FWHM) bandwidth of the MPF is 192 MHz, indicating a Q factor of 1.01 × 10^6^. The extinction ratio is about 19 dB. The center frequency of the single bandpass MPF shown in Fig. 5 is around 10 GHz, which is determined by the wavelength difference between the optical carrier and the − 1st-order sideband. As the carrier is at the middle of two adjacent resonances of MRR, another passband of the MPF will be generated by the adjacent resonance of the MRR with a center frequency of 16 GHz, based on the same principle. The sum of the center frequencies of the two passbands is about 26 GHz, corresponding to the FSR of 0.209 nm of the MRR. To keep the unwanted passband out of the measuring range, the tuning range of the center frequency should be smaller than half of 26 GHz. During the following experiment, the tuning range of the temperature is set to be 1.0 °C and the measuring range of the frequency is set as 12 GHz. By connecting the two straight multi-mode waveguides with multi-mode Euler curves instead of single-mode waveguides and tapers, the perimeter of the ring can be reduced while keeping low transmission loss. The FSR of the MRR can be increased and the temperature sensing range of the proposed sensor can be improved while the Q factor of the MRR can remain higher than a million [28].

**Fig. 6:**
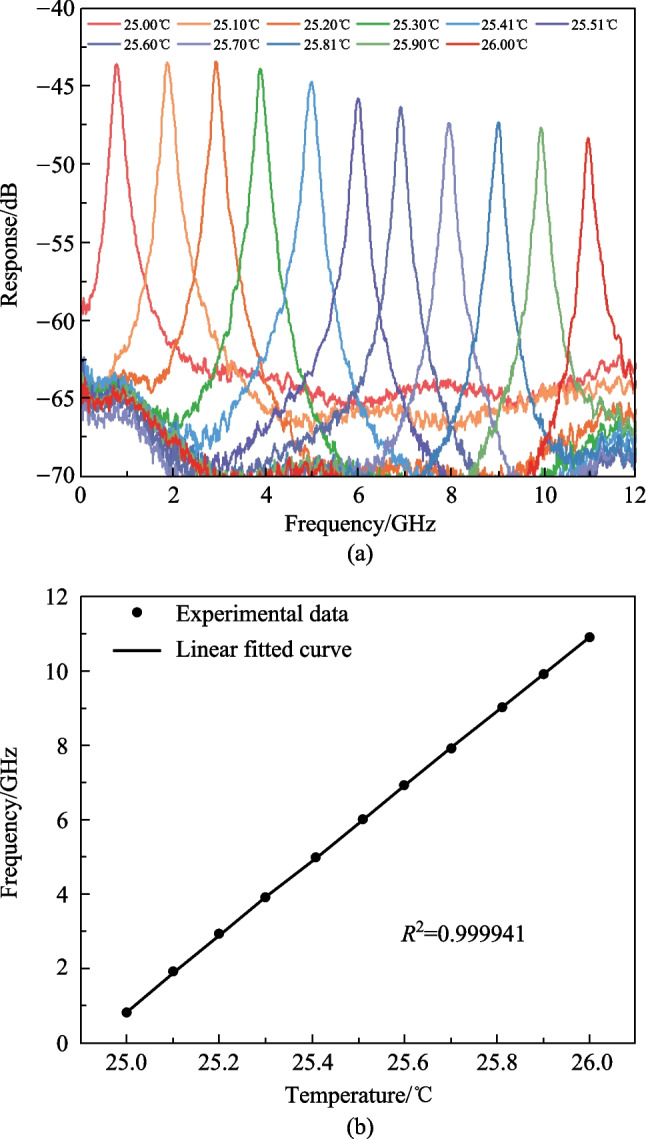
Experimental results of the proposed MPF-based temperature sensor. **a** Measured responses of the MPF at different temperatures. **b** Measured center frequencies of the MPF versus temperature and the linear fitted curve of the experimental data

To verify the performance of the proposed MPF-based temperature sensor, the relationship between the center frequency of the MPF and the temperature of MRR is measured. By changing the temperature of the TEC, the monitored temperature of the MRR changes as well. The measured responses of the MPF at different temperatures are shown in Fig. 6a. As the temperature changes from 25.00 °C to 26.00 °C with a step of 0.10 °C, the center frequency of the MPF changes from 0.89 to 10.94 GHz with a step of about 1.00 GHz. The center frequency versus temperature plot is given in Fig. 6b. The linear fitted curve shows the slope, which indicates the sensitivity, is 10.08 GHz/°C and the coefficient of determination (*R*^2^) is 0.999941.

**Fig. 7:**
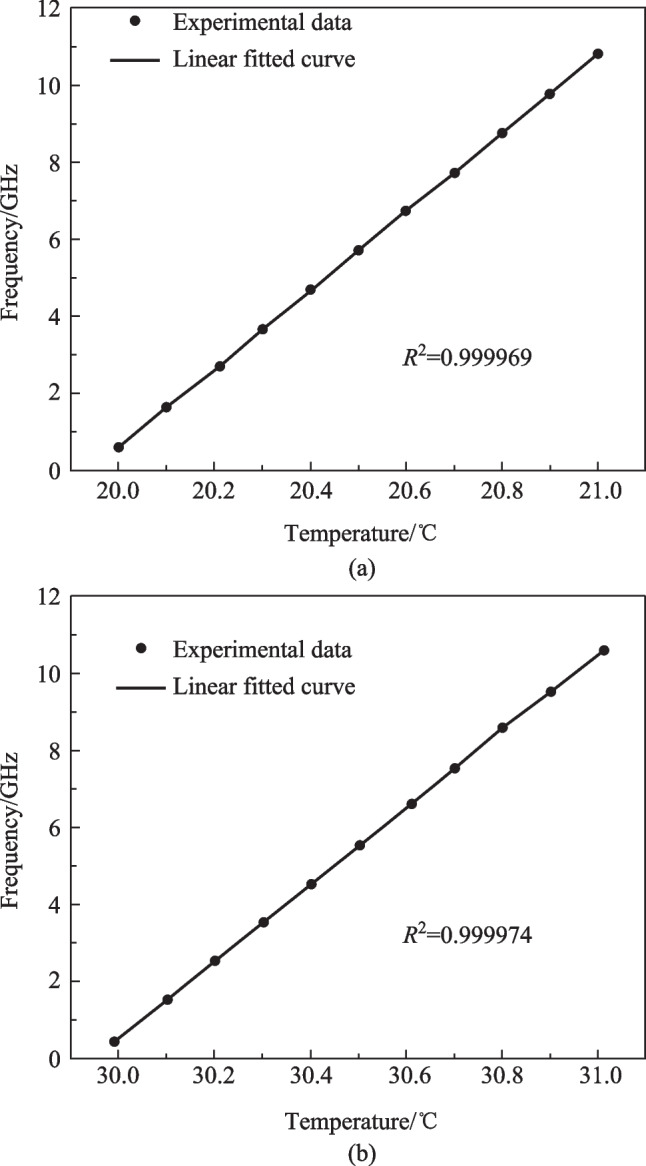
Measured center frequencies of the MPF versus temperature and the linear fitted curve of the experimental data near the temperature of **a** 20 °C and **b** 30 °C

The performances of the proposed MPF-based temperature sensor near 20 °C and 30 °C are also investigated. When the temperature changes from 20.00 °C to 21.00 °C and from 29.99 °C to 31.01 °C, the measured center frequency versus temperature plots are shown in Fig. 7a, b, respectively. The linear fitted curves show the slopes are 10.22 and 9.97 GHz/°C while the coefficients of determination (*R*^2^) are 0.999969 and 0.999974 around the temperatures of 20 °C and 30 °C, respectively. In Fig. 7a, b, the difference in *R*^2^ is caused by the measuring error of the temperature, which is inevitable in the experiment since the controlling resolution of the TEC is limited to 0.01 °C. Because of the high spectral resolution brought by the MRR of ultra-high Q factor as well as the high sensitivity brought by the silicon-based device, the sensing resolution of the proposed temperature sensor is as high as 0.019 °C.

## Conclusion

An MPF-based temperature sensor has been proposed and successfully demonstrated. The MPF has a simple configuration, mainly consisting of a laser source, a PM, an MRR, and a PD, which has the potential to be monolithically integrated. The SOI-based MRR is designed with multi-mode ridge waveguides to decrease propagation loss. Consequently, an ultra-high Q factor of 1.01 × 10^6^ is experimentally demonstrated. A single passband MPF with a narrow bandwidth of 192 MHz is achieved and the sensor performance is validated at temperatures around 20 °C, 25 °C and 30 °C respectively, within a variation range of 1 °C. The sensitivity of the proposed temperature sensor is as high as 10.22 GHz/°C. As a result of the high sensitivity and narrow bandwidth of the MPF, the sensing resolution of the proposed temperature sensor is as high as 0.019 °C.

## Data Availability

The data that support the findings of this study are available from the corresponding author, upon reasonable request.
